# Selected anti-tumor vaccines merit a place in multimodal tumor therapies

**DOI:** 10.3389/fonc.2012.00132

**Published:** 2012-10-09

**Authors:** Eva-Maria Weiss, Roland Wunderlich, Nina Ebel, Yvonne Rubner, Eberhard Schlücker, Roland Meyer-Pittroff, Oliver J. Ott, Rainer Fietkau, Udo S. Gaipl, Benjamin Frey

**Affiliations:** ^1^Department of Radiation Oncology, University Hospital Erlangen, Friedrich-Alexander-Universität Erlangen-NürnbergErlangen, Germany; ^2^Department of Process Technology and Machinery, Friedrich-Alexander-Universität Erlangen-NürnbergErlangen, Germany; ^3^Competence Pool Weihenstephan, Technische Universität MünchenFreising, Germany

**Keywords:** immunotherapy, vaccination, cancer therapy, multimodal, anti-tumor immunity, whole cell-based vaccines, high hydrostatic pressure, radiotherapy

## Abstract

Multimodal approaches are nowadays successfully applied in cancer therapy. Primary locally acting therapies such as radiotherapy (RT) and surgery are combined with systemic administration of chemotherapeutics. Nevertheless, the therapy of cancer is still a big challenge in medicine. The treatments often fail to induce long-lasting anti-tumor responses. Tumor recurrences and metastases result. Immunotherapies are therefore ideal adjuncts to standard tumor therapies since they aim to activate the patient's immune system against malignant cells even outside the primary treatment areas (abscopal effects). Especially cancer vaccines may have the potential both to train the immune system against cancer cells and to generate an immunological memory, resulting in long-lasting anti-tumor effects. However, despite promising results in phase I and II studies, most of the concepts finally failed. There are some critical aspects in development and application of cancer vaccines that may decide on their efficiency. The time point and frequency of medication, usage of an adequate immune adjuvant, the vaccine's immunogenic potential, and the tumor burden of the patient are crucial. Whole tumor cell vaccines have advantages compared to peptide-based ones since a variety of tumor antigens (TAs) are present. The master requirements of cell-based, therapeutic tumor vaccines are the complete inactivation of the tumor cells and the increase of their immunogenicity. Since the latter is highly connected with the cell death modality, the inactivation procedure of the tumor cell material may significantly influence the vaccine's efficiency. We therefore also introduce high hydrostatic pressure (HHP) as an innovative inactivation technology for tumor cell-based vaccines and outline that HHP efficiently inactivates tumor cells by enhancing their immunogenicity. Finally studies are presented proving that anti-tumor immune responses can be triggered by combining RT with selected immune therapies.

## Immunotherapy in cancer treatment

Today's cornerstones in cancer therapy are radiotherapy (RT), chemotherapy (CT), and surgery. Local tumor control and/or complete regression are achievable for many tumor entities, since these methods alone and especially combinations of them have been further improved. Nevertheless, development of tumor recurrence and metastases substantially deteriorates the patient's prognosis. To win the fight against cancer is therefore not only restricted to kill all tumor cells of the primary tumor, but also to act on the patient's whole body to achieve a long-lasting anti-tumor effect which keeps remaining and recurrent tumor cells in check. Therefore, systemic approaches are required that activate the patient's immune system against the tumor. The cells of the immune system have to be trained to control residual disease and hidden metastases (Sistigu et al., 2011).

Cancer immunotherapies (CI) aim to be, beneath their role in primary local tumor killing, the second line therapy against recurrent tumors and metastases by priming the patient's immune system to elicit an anti-tumor response (Sharma et al., 2011). First and foremost, the combination of standard therapies with immunotherapeutic strategies is auspicious to reach stable disease and to improve overall survival. Additionally, due to a lower toxicity compared with chemotherapeutic agents, immunotherapeutic approaches are more compliant for normal tissue and the whole organism. Especially combinations of RT and CI are promising since they are capable to elicit anti-tumor effects outside the radiation field, a phenomenon called abscopal effect (Demaria et al., 2004; Frey et al., 2012). RT with ionizing irradiation (X-ray) triggers the release of pro-inflammatory signals. It further indirectly contributes to the activation of dendritic cells (DCs) by generating modified and new tumor specific antigens and by inducing the release of danger signals of irradiated tumor cells. Further, an enhanced loading of DCs with tumor antigens (TAs) of irradiated tumor cells has been observed (Teitz-Tennenbaum et al., 2008). Therefore, combination of RT with DC-based CI is considered to be ideal for the induction of tumor specific immune responses.

**Figure 1:**
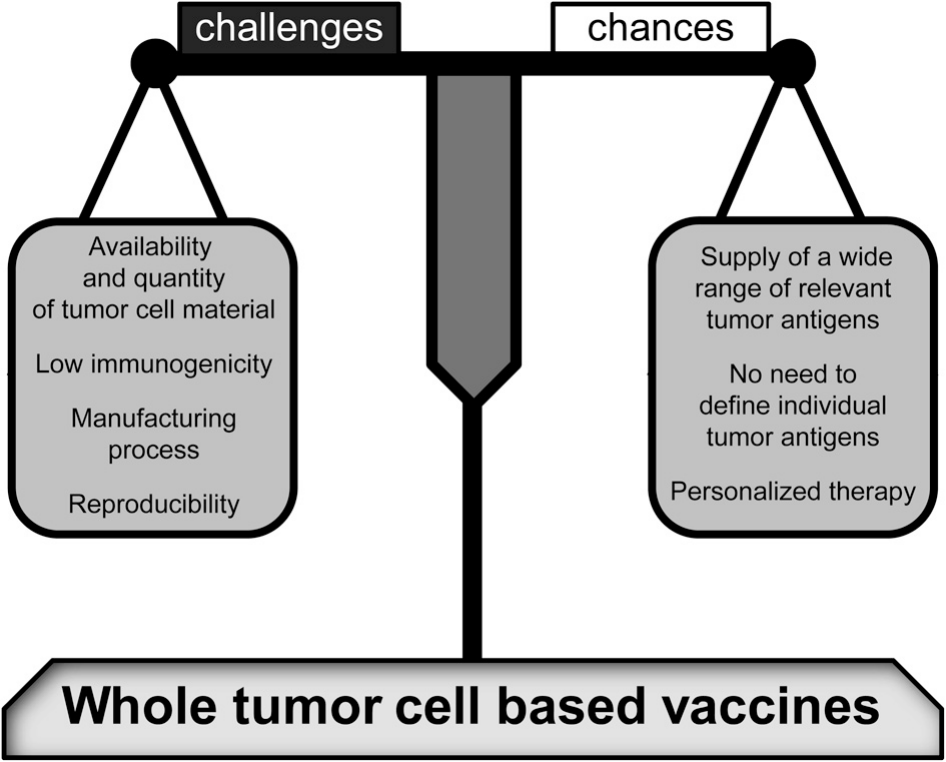
**Challenges and changes of whole tumor cell-based vaccines.** As compared to peptide-based vaccines, a high variety of relevant tumor antigens is provided by whole tumor cell-based vaccines. Therefore, the tumor antigens have not to be individually defined. However, there are some crucial challenges that may decide about the vaccine's efficiency and productivity including the immunogenic potential of tumor cell material and a reliable manufacturing process associated with a high reproducibility. Furthermore, the limited availability of tumor cell material has to be taken into account. Nevertheless, when these resolvable problems have been resolved, whole tumor cell-based vaccines will be prime immune therapies for personalized tumor treatments.

The field of immunotherapy offers a broad array of approaches including monoclonal tumor specific antibodies that have been already established in clinical treatment of several tumor entities (Scott et al., 2012), the application of immune activating cytokines (Dranoff, 2004), or even gene transfer of adoptive T-cell receptor (TCR) to obtain large numbers of tumor-reactive T cells. The *in vivo* stimulation of an anti-tumor response by vaccines is another important approach. Especially whole tumor cell-based vaccines offer a wide array of TAs. Contrary to peptide-based vaccines, defining and manufacturing of individual and immunogenic antigens is not required since whole cells comprise all immunologically relevant tumor peptides (Figure 1). Of special note is that this multiplicity decreases the risk of tumor escape.

Crucial in generating effective whole tumor cell vaccines is to induce, or even increase their immunogenicity (Frey et al., 2008). Since the way cells die is closely connected to their immunogenic potential, the inactivation process of tumor cells is often the determining factor for a vaccine's potency (Tesniere et al., 2008a,b). Currently, we investigate high hydrostatic pressure (HHP, meaning pressure stages >100 MPa) treatment as a novel inactivation technology of whole tumor cells. We already proved that various tumor cell lines can be efficiently inactivated by treating them with pressure ≥200 MPa and observed in preclinical mouse models that that HHP-killed tumor cells are immunogenic (Weiss et al., 2010b).

## Immune therapies with cytokines and monoclonal antibodies

Before we go into detail how whole tumor cell vaccines induce anti-tumor immunity, we will shortly introduce further strategies of CI with “agents” that do not bear tumor peptides and antigens such as cytokines or monoclonal antibodies.

Cytokines in the tumor microenvironment have a strong influence on the host's immunity. They may foster or suppress tumor growth (Chometon and Jendrossek, 2009; Apte, 2010). Consequently, the administration of distinct cytokines in cancer therapy can modulate the microenvironment of a tumor in a way that leads to a better therapeutic outcome (Dranoff, 2004). However, their administration can also induce relevant side effects related with a moderate effectiveness (Kelley et al., 2003; Dantzer and Kelley, 2007). Hence, combination of cytokines with other strategies allows dose reduction. Clinically successful phase III trials have been carried out with systemic administration of interleukin (IL)-2, that enhances natural killer (NK)-cell and T-cell activity (Rosenberg et al., 1993; Fyfe et al., 1995), or stimulators for TA presentation like granulocyte-macrophage colony-stimulating factor (GM-CSF) (Dranoff et al., 1993), interferon (IFN)-α (Biron, 2001), or IFN-γ (Bach et al., 1997). Since immunity against cancer is a multi-step-process, the sole application of cytokines is insufficiently. The role of cytokines in cancer therapy and pathogenesis has been extensively discussed during the last years (Dranoff, 2004; Margolin, 2008; Mellman et al., 2011).

Beyond, immunity against malignant cells can be established with monoclonal antibodies that trigger tumor cell apoptosis by inducing antibody-dependent cellular or complement-mediated cytotoxicity. Further, those antibodies may block growth factor receptors or foster anti-tumor immune responses (reviewed in King et al., 2008; Scott et al., 2012; Weiner et al., 2012).

Rituximab, an antibody that targets CD20 on B-cells and causes B-cell apoptosis in B-cell lymphoma (Pescovitz, 2006), is one of the prominent examples for the application of monoclonal antibodies in CI. Others are antibodies such as Trastuzumab (Hudis, 2007; Valabrega et al., 2007), acting against human epidermal growth factor receptor 2 (HER-2) on cancer cells, or Cetuximab, that acts against the epidermal growth factor receptor (EGFR) (Cunningham et al., 2004; Bonner et al., 2006). Bevacizumab is directed against vascular endothelial growth factor (VEGF) and the anti-cytotoxic T-lymphocytes (CTL) A-4 antibody Ipilimumab abrogates the inhibitory effects of CTLA-4 on T-cell activation (Greenwald et al., 2001; Ferrara et al., 2004; Scott et al., 2012). Via binding to Fc gamma receptors (FcγR) on DCs, monoclonal antibodies contribute further to an induction of adaptive anti-tumor immune responses. An antibody-mediated enhanced cross-presentation of TAs was observed (Dhodapkar et al., 2002, 2005; Weiner et al., 2010). Similar to chemotherapeutics, monoclonal antibodies act directly in the whole tumor mass, but are more compliant for the patient, due to their low toxicity. However, monoclonal antibodies have a narrow target antigen range. Nevertheless, a plenty of studies have been performed using such antibodies. Scott and colleagues recently summarized the advantages and disadvantages of this therapeutic option against cancer (Scott et al., 2012).

Besides the above mentioned mechanisms, the potential of antibodies and cytokines to activate the immune system accompanied with an establishment of immunological memory is still low. A combination of CI strategies might be beneficial to induce anti-tumor immunity and reduce the tumor cell growth and induce tumor cell death, respectively (Van Elsas et al., 1999; Takaku et al., 2010; Weiss et al., 2010a).

## Immunological basics of the mode of action of tumor vaccines

Contrary to monoclonal antibodies, tumor vaccine strategies aim to actively train the immune system going along with the development of a long-lasting immunological memory. Hence, particularly the appearance of metastases and tumor recurrences can potentially be counteracted or even avoided. Both, the innate and the adaptive arm of the cellular immune system can contribute to an effective attack toward tumor cells.

Lymphokine-activated killer (LAK) cells, NK cells, and macrophages are crucial players of innate immunity, acting with a low specificity but widespread against tumor cells with different histological background (Grimm et al., 1982; Rosenberg et al., 1985; Krause et al., 2002; Terme et al., 2008). Cells of the adaptive immune system, comprising CD4+ and CD8+ T-lymphocytes, are more suitable to elicit a long-lasting immune response, since they can specifically target TAs and differentiate to memory cells. The three most important processes leading to long-lasting tumor immunity were recently proposed by Mellman et al. (2011). In the first step, DCs must sample relevant TAs. Secondly, DCs have to mature and to initiate the T-cell response. Last but not least, the T-cells have to overcome the immunosuppression of the solid tumor and enter the tumor bed (Mellman et al., 2011).

Tumor vaccinations offer a variety of strategies to activate and train the immune system. Vaccination with distinct TAs, or whole tumor cells have been performed to activate DCs *in vivo.* Further, strategies with pulsing of DCs *ex vivo* with TAs are followed up. Nevertheless, the generation of an effective, specific, and long-lasting response against tumor cells is more challenging compared to that against pathogens, since the tumor had repeatedly escaped an immune surveillance (Novellino et al., 2005).

Transformed cells have several strategies to circumvent immune activation (reviewed in Dunn et al., 2002; Igney and Krammer, 2002). For example, presentation of TA on the tumor cells' surface is generally poor. This is even more present in metastases that are often characterized by frequent mutations; this helps transformed cells to escape from an initially induced specific response (Kim et al., 1975; Bailly et al., 1993). Moreover, presentation and protein loading of major histocompatibility complex (MHC)-class I molecules is often reduced or malfunctioned avoiding lysis by cytotoxic T cells (Hicklin et al., 1999). Further mechanisms in the microenvironment contribute to tumor escape, such as the secretion of immunosuppressive factors including TGF-β (Li et al., 2006), IL-10 (Wittke et al., 1999), or prostaglandin (Botti et al., 1998). These cytokines secreted by tumor cells favor an immune deviation, characterized by a shift toward a more Th2-polarized response that suppresses the establishment of an adaptive cellular response involving effector CD8+ T-lymphocytes (Maeda and Shiraishi, 1996; Shurin et al., 1999; Ribas et al., 2000). The cytokine profile, a low stimulation by TA and the missing stimulative conditions can finally lead to T-cell anergy, to the induction of regulatory T-cells, and also to T-cell-depletion (Staveley-O'Carroll et al., 1998; Zou, 2006). The major challenges of an effective cancer vaccine are therefore to overcome these immune suppressing mechanisms and to train the immune system to recognize and to attack tumor cells.

For the induction of an effective and long-lasting anti-tumor response, priming of CTL is crucial. CTLs are able to specifically target TAs and to destroy tumor cells directly. Moreover, they may differentiate into memory T-cells, which enable the development of a prolonged anti-tumor response (Podack, 1995; Shresta et al., 1998). For activation, T-cells have to meet their specific TA and additionally have to receive co-stimulatory signals. Recognition of TA is mediated by TCRs on naïve T-lymphocytes and antigen-MHC complexes on antigen presenting cells (APCs). The binding of the TA is restricted to MHC-class I or MHC-class II molecules on CD8+ and CD4+ T-cells, respectively. Furthermore, APCs provide co-stimulatory signals, including B7.1/B7.2 molecules that interact with CD28 on T-cells, or CD40 receptors on T-cells interacting with CD40 ligand (CD40 L). Without those additional signals T-cells are not able to proliferate and to produce cytokines; they even undergo apoptosis (Frauwirth and Thompson, 2002). Contrary to the activation of CD4+ T-cells, the priming of CTLs from naïve CD8+ T-cells is more complex and involves the interplay of DCs, T helper cells type 1 (Th1 cells), and cytokines (Mellman et al., 2011).

Immature DCs constantly migrate through tissues and blood, scanning their environment for potential pathogens or danger signals. DCs recognize invading pathogens with their pathogen-associated molecular patterns (PAMPs) recognition receptors (PRRs). But DCs are not only activated by pathogen-derived signals. According to the danger theory of Polly Matzinger (Matzinger, 1994), the immune system is able to distinguish between danger and non-danger. Dying mammalian cells release danger-associated molecular patterns (DAMPs) that act as potent stimuli for DCs. In the last years several DAMPs have been described, including high mobility group box 1 protein (HMGB-1), heat shock proteins (HSPs), and uric acid (Shi et al., 2003; Bianchi, 2007). After the recognition by DCs, the tumor cell is engulfed and antigen processing takes place. Maturation of DCs has been initiated and is accompanied by a decreasing potential of antigen assimilation combined with increased migration ability. Consecutively, DCs migrate to the lymph node (LN), where their peptide/MHC-class II complex is presented to the antigen specific TCR on naïve CD4+ T-cell. DCs have to reach a fully mature stage to power an effective immune response, because semi-mature DCs have rather tolerogenic features (Rutella et al., 2006; Mellman et al., 2011).

The fate of T helper cell subtypes and therefore the type of immune response are strongly determined by polarizing factors that are secreted by DCs (O'Garra and Arai, 2000). The cytokines IL-2, IL-12, and IFN-γ favor the differentiation of naïve CD4+ T-cells to T helper cell type 1 (Th1), while IL-10, IL-4, and IL-5 polarize a T helper cells type 2 (Th2) response (O'Garra and Arai, 2000). In regard to a CTL-mediated tumor response, the induction of a Th1 response is crucial since the interaction of Th1 cell with DCs renders the DCs themselves capable for the activation of naïve CD-8+ T-cells by CD40 and CD40-L interaction (Schoenberger et al., 1998). Consequently, the primary contact between tumor cells and DCs is pivotal for both the initialization and the polarization of adaptive immune responses.

The cytokine milieu may also favor several other T helper subclasses besides Th1 and Th2 cells. For example, the development of Th17 cells that secrete the cytokine IL-17 is promoted by TGF-β and IL-6. The role of Th17 cells in tumor progression and/or regression is under current investigation (Langowski et al., 2006; Hirota et al., 2012; Liu et al., 2012; Middleton et al., 2012). In conclusion, the course of adaptive immunity against cancer cells is already initialized by innate immune cells, in particular by DCs. The priming of DCs by malignant cells is mainly determined by microenvironmental factors of the “meeting point” (Kapsenberg, 2003; De Jong et al., 2005).

Since danger signals released by dead tumor cells dictate the DC's behavior, they are crucial for the induction of anti-tumor immune responses. Toll like receptors (TLR) on the DCs' surface can recognize both PAMPs and DAMPs and have a great impact on anti-tumor immunity (Tesniere et al., 2008a). It has been reported that ligation of TLR-4 and TLR-5 instructs DCs to stimulate naïve T helper cells to a Th1 polarization while binding of TLR-2 dictates a Th2 response (Agrawal et al., 2003). DAMPs that are released by dying and dead cells can bind to TLRs and are therefore capable to determine priming of DCs. For example, HMGB-1 favors binding to TLR-4, while HSP-70 can interact with TLR-2 and TLR-4 (Apetoh et al., 2007; Asea, 2008). Hence, the form of tumor cell death, induced either *in vivo* by direct cytotoxic therapy or *ex vivo* for vaccination purpose, strongly determines whether an anti-tumor immune response is elicited or not (Tesniere et al., 2008a). Inactivation technologies for the preparation of whole tumor cell vaccines should therefore aim to induce immunogenic tumor cell death forms.

## Cell death forms an immunological relevance

Today a variety of different cell death forms is described for mammalian cells. Some cell death forms are highly genetically determined; others display more accidental characteristics (Griffith and Ferguson, 2011). Most attention was given to the two main cell death forms, namely apoptosis and necrosis, since their immune modulatory potential helped to understand how chronic autoimmune diseases might develop and/or sustained (Gaipl et al., 2006; Gaipl, 2009). Usually, apoptotic cells are immunologically silent or even tolerogenic. They are part of a physiological process to maintain homeostasis of every multicellular organism. Apoptosis is characterized by several cell morphological and biochemical features like DNA fragmentation, cell blebbing, and condensation of the chromatin (Kerr et al., 1972; Griffith and Ferguson, 2011). The silent clearance of apoptotic cells is mediated by “find-me” signals that are released by apoptotic cells to promote the attraction of phagocytes (Lauber et al., 2004; Ravichandran and Lorenz, 2007). The latter recognize “eat me” signals on the apoptotic cells' surface, leading under healthy conditions to a swift clearance of the dying cells. Engulfment of apoptotic cells provokes in activated phagocytes even the secretion of anti-inflammatory signals such as IL-10 and TGF-β (Voll et al., 1997). However, it has recently turned out that under certain circumstances apoptosis may also exhibit immune-stimulatory features, in particular when treated with certain chemotherapeutics (anthracyclines) or γ-irradiation (Casares et al., 2005; Obeid et al., 2007; Tesniere et al., 2008a; Locher et al., 2010). The associated molecular mechanisms are not yet fully investigated, anyhow there is evidence that the early exposition of the ER resident chaperone calreticulin together with ERP57 on the cell surface is one key part of it (Obeid, 2008; Ma et al., 2011).

Contrary to the predominantly immunologically silent manner of apoptotic cells, necrosis is associated with (pro-) inflammation; hence it's conditioned by a pathological process (Golstein and Kroemer, 2007). The loss of membrane integrity results in the secretion of danger signals that may lead to the activation and maturation of immune cells and generally generates inflammatory conditions. Extracellular HSPs (Asea, 2008; Schmid and Multhoff, 2012) and HMGB-1 are prominent examples for such released immune activator proteins (Apetoh et al., 2007; Bianchi, 2009; Schildkopf et al., 2011). Interestingly, some danger signals can be released by necrotic as well as apoptotic cells. One has to consider that in the case of apoptosis the danger signals are often modified before their release resulting in a contrary immunological outcome (Griffith and Ferguson, 2011). For example, HMGB-1 is usually oxidized by reactive oxygen species during the apoptotic process and thereby loses its immunological potency (Urbonaviciute et al., 2009). This highlights that the dying cell itself and its microenvironment determines whether immune activation or immune suppression is triggered. The different forms of cell death are manifold and sometimes hard to differentiate. Garg and colleagues recently summarized what factors determine the immunogenicity of the dying cells (Garg et al., 2010). Even a programmed form of necrosis, the so called necroptosis, has been described and the immunological consequences of this cell death modality are currently under investigation (Galluzzi et al., 2012).

If whole tumor cell vaccines are prepared by inactivation of tumor cells, the immunogenicity of the dead cells should be enhanced or at least maintained by this procedure. A main focus of future cancer therapy concepts should be set on combination of classical anti-cancer therapies with immunotherapy in order to accomplish the anti-tumor effects of both concepts. Standard therapies such as RT or the treatment with particular chemotherapeutics should induce highly immunogenic dead cells which generate an immune activating microenvironment (Ma et al., 2011). Solely immunotherapeutic agents are not sufficient to efficiently shift a strong anti-inflammatory tumor microenvironment to an inflammatory one. Therefore, the tumor cell death form of the whole tumor cells used as vaccine as well as the cell death induced in the primary tumor by standard therapies are of great importance to trigger an effective anti-tumor immunity.

## Autologous and allogeneic whole tumor cell-based vaccines in the clinic

During the last years, several vaccination strategies were evaluated in pre-clinical and clinical phase I and II studies. However, most of the tested vaccination approaches finally failed to achieve clinical success in randomized phase III trials (summarized in Rosenberg et al., 2004; Itoh et al., 2009; Klebanoff et al., 2011). Table 1 summarizes the phase III trials containing whole tumor cell-based vaccines. In 2010, an autologous DC-based vaccine (Sipuleucel-T), used in prostate cancer, achieved FDA approval in the USA (Cheever and Higano, 2011) and has led to a boost in the development of new vaccination strategies and agents. For an improvement of vaccine concepts, the big challenge for research is to figure out the reasons for previous clinical failings of the vaccines.

**Table 1:** **Overview of whole tumor cell-based vaccines that have been tested in Phase III trials**.

**Trade name**	**Type of vaccine**	**Cancer type**	**Phase**	**Included patients**	**Observation periode**	**Reference**
Sipuleucel-T	Autologous dendritic cell-based vaccine incubated with PAP-GM-CSF fusion protein	Prostate cancer	III	512	3 years	Cheever and Higano, 2011
Reniale	Cell-lysate-based autologous vaccine by freeze/thaw cycles	Renal-cell carcinoma	III	379	5 years	Jocham et al., 2004
Reniale	Cell-lysate-based autologous vaccine by freeze/thaw cycles	Renal-cell carcinoma	III	692	10 years	May et al., 2010
Oncovax	Irradiated autologous tumor cell-based vaccine with BCG	Colorectal cancer	III	317	5 years	Simons and Sacks, 2006
Prostate-GVAX	Allogenic cell-based, GM-CSF gene transduced vaccine	Prostate cancer	III	408	Prematurely terminated	Higano et al., 2009
Prostate-GVAX	Allogenic cell-based, GM-CSF gene transduced vaccine	Prostate cancer	III	626	Prematurely terminated	Small et al., 2009
Canvaxin	Irradiated allogenic cell mix-based vaccine with BCG	Melanoma	III	Stage III: 1100	Prematurely terminated	Finke et al., 2007
Canvaxin	Irradiated allogenic cell mix-based vaccine with BCG	Melanoma	III	Stage IV: 670	Prematurely terminated	Finke et al., 2007

Most of the clinical studies reviewed by Rosenberg and Klebanoff applied strategies based on vaccination with a single peptide or protein. These vaccines have the disadvantage to be restricted to a single target or, in the case of proteins, to few epitopes. Identification of TAs and proof of their immunogenicity are challenging aspects. Difficulties may result from poor antigen presentation on tumor cells or the appearance of frequent mutations in metastases: Both may result in the loss of peptide-specific effector T-cells' ability to recognize the tumor cells. Additionally, the efficiency of peptide-based vaccines is mostly HLA-restricted, resulting in a constricted quantity of potentially responding individuals (Chiang et al., 2010). Whole tumor cell vaccines are promising to bypass complex procedures in defining individual antigens. The tumor cell surface comprises a huge amount of potentially relevant antigens. Besides, the provided antigen plurality impedes tumor escape. For an enhanced anti-tumor response the additional application of immune adjuvants such as Bacillus Calmette-Guérin (BCG) or cytokines is beneficial. *Ex vivo* genetic manipulations of inactivated cell material are also followed up, resulting in the secretion of GM-CSF (Simons and Sacks, 2006), other cytokines, chemokines, or in increased expression of MHC antigens (Pardoll, 1995).

Autologous tumor cell-derived material has already been demonstrated in clinical studies to be a promising cancer vaccine. An adjuvant renal tumor cell lysate-based vaccine (Reniale®) was applied in a phase III study for patients with renal-cell carcinoma after nephrectomy. The 5-year progression-free survival rate improved to 77.4% compared to 67.8% in the control arm (Jocham et al., 2004). In another study, a ten-year survival analysis was performed for renal carcinoma patients also treated with that autologous tumor lysate vaccine in an adjuvant setting. An overall survival benefit (OS rates of 68.9% in the study group versus 62.1% in the control group) was observed. Especially the subgroup of patients with pT3 stage tumors did profit from this adjuvant vaccination (OS rates of 53.6% in the study group versus 36.2% in the control group) (May et al., 2010). For those vaccinations, necrotic cell lysates were obtained by freeze/thaw cycles of whole tumor cells. These lysates consist of cell fragments including parts of cellular membrane, RNA, and DNA as well as cell organelles. Since the cell membrane is disturbed, danger signals such as HSPs and HMGB-1 are released and may *in vivo* stimulate the maturation of DCs (Sauter et al., 2000; Somersan et al., 2001). Until today no generalized vaccination recommendations for patients with renal cancer are given since further controlled trials are recommended and necessary. Furthermore, it has become obvious, that subgroups of patients exist that might benefit most from such vaccinations (May et al., 2010).

For colorectal cancer, a vaccine consisting of irradiated whole autologous tumor cells (Oncovax®) has been evaluated in clinical studies (up to phase III) (Hanna Jr et al., 2001; Simons and Sacks, 2006). For immunotherapy of melanoma, a quite similar approach was tested. Patients diagnosed with metastatic melanoma stage III/IV (*n* = 81) were included. After resection, melanoma cell material was irradiated and re-injected together with BCG. A survival benefit of the patients who received the vaccine was observed but it was only restricted to patients without evidence of macroscopic disease during the vaccination period (Baars et al., 2000). The delayed type hypersensitivity response (DTH) correlated with the survival data of the patients.

These clinical trials illustrate that despite disappointing clinical results of cancer vaccines (Klebanoff et al., 2011) also positive ones exist. However, the clinical studies with whole tumor cell vaccines curtailed the high expectations that were put into them. Nevertheless, whole tumor cell-based vaccines have to be brought back to bench and then again back to bedside, because highly immunogenic autologous tumor cell-based vaccines may offer great changes to cure cancer, especially when used in an adjuvant setting to avoid tumor recurrences or metastases.

Besides autologous tumor cells, allogeneic ones might also be used as cancer vaccines. Autologous tumor cells have the advantage to provide the complete set of personalized TAs; including individual mutated ones (Fournier and Schirrmacher, 2009). Anyhow, relevant drawbacks exist with regard to the production of autologous cell vaccines. Resectable tumors are needed to obtain enough tumor material. The number of tumor cells obtained after resection is often limited, especially in the case of metastases or early stage tumors. To obtain an adequate tumor cell amount, *in vitro* cultivation and expansion is often required entailing other difficulties (e.g., maintenance of cell integrity, prolonged process duration) that may influence the immunogenicity of the vaccine (Copier et al., 2007).

GVAX technology is one example for vaccine development where genetically modified tumor cells were used for vaccination. Granulocyte macrophage colony-stimulating factor (GM-CSF) is transduced into the tumor cells since it has proven immune stimulatory properties. It promotes recruitment and maturation of DCs and further up-regulation of MHC-class II molecules, co-stimulatory molecules, and cytokine production in DCs. Both, preclinical trials in a melanoma model (Dranoff et al., 1993) and a clinical studies in melanoma patients have shown the potential of autologous GVAX cells to elicit a tumor specific long-lasting anti-tumor response (Hege et al., 2006). GVAX was tested in numerous tumor entities with autologous tumor cell lines including renal (Tani et al., 2004), melanoma (Kusumoto et al., 2001; Soiffer et al., 2003), and non-small lung carcinoma (Salgia et al., 2003). Finally, GVAX therapies were designed for allogeneic settings in pancreatic and prostate cancer (Copier et al., 2007). “Prostate-GVAX” is the most advanced approach and two randomized phase III studies have been running (Higano et al., 2009; Small et al., 2009). The vaccine consists of two prostate cancer cell lines that were genetically modified by adenoviral transfection to produce GM-CSF. In one trial, GVAX was compared with CT (docetaxel plus prednison), while the other one comprised GVAX plus CT as study arm, and CT alone as control arm. Disappointingly, both studies were terminated prematurely, because it has turned out that control arm was more beneficial compared to the study arm regarding OS (Joniau et al., 2012).

There are further approaches using allogenic, genetically modified whole tumor cells as tumor vaccine. The group of Nemunaitis et al. tested the administration of the TGF-beta2 antisense gene modified allogeneic tumor cell compound in the treatment of non-small lung carcinoma; promising results were obtained in a phase II trial (Nemunaitis et al., 2006). The administration of the vaccine resulted in partial response rates of 15%, but failed in initiating a significant improvement in the overall response rate in the vaccine group. It was therefore not pursued in clinical trials (Nemunaitis et al., 2006). Contrary, canvaxin, as an example for a whole cell tumor vaccine consisting of unmodified allogeneic tumor cells, made its way into clinical trials. Canvaxin is composed of irradiation inactivated three different cell lines that express various TAs for melanoma. Administrated with BCG as an adjuvant resulted in immunological responses detected by DTH response and IgM level in the sera of the patients. This response correlated with increased median survival (Morton et al., 1992). The canvaxin phase II studies were promising and had proven survival benefits for stage III melanoma patients and even complete remissions in patients with low volume disease (after surgical removal) (Hsueh et al., 1999; Morton et al., 2002). Nevertheless, this approach failed in a phase III study, which was terminated earlier because the efficiency of control arm (BCG + placebo) was stronger, compared to the study arm (Finke et al., 2007). To conclude, many of the allogeneic-based vaccine approaches failed in the end in clinical trials. Therefore, autologous tumor cell based vaccines will be more in the focus of future vaccine developments (Fournier and Schirrmacher, 2009).

## High hydrostatic pressure as promising vaccine preparation method

Although there are some promising approaches in the field of whole cell-based vaccines, the clinical outcome still remains unsatisfactory. One major problem could be the low immunogenicity of the vaccines and/or the failure of establishing an immunological memory. The clinical success is mainly proven in phase I and phase II trials. Dalgleish suggests that differences in clinical centers may contribute to the disappointing outcome in the phase III randomized studies and assumes that parameters like diet, exercise, and supplements might determine anti-tumor effect more than supposed (Dalgleish, 2011).

The production of the vaccines is another major challenge. Long processing times and reproducibility are still major problems. Strategies are needed that allow both, simple processing of tumor cells by concomitantly rendering them immunogenic. Cell death pathways and death stimuli are closely connected to the tumor cell's immunogenic potential. However, despite the numerous vaccine approaches, hardly any work has taken into account the immunogenicity of the tumor cells that is determined by the different inactivation methods (Tesniere et al., 2008a).

**Figure 2:**
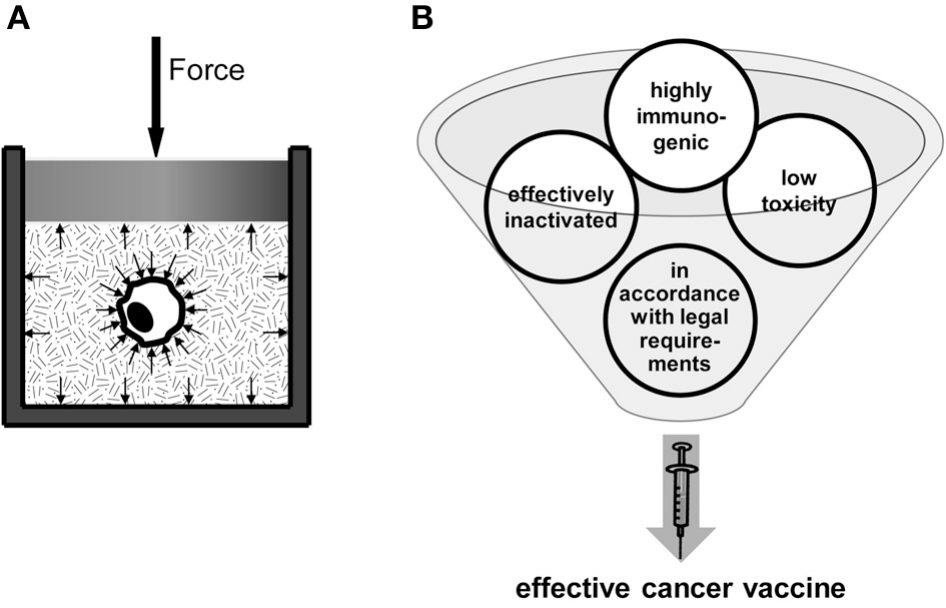
**High hydrostatic pressure (HHP) Technology as an innovative method for the preparation of whole tumor cell-based vaccines.** The basic principle of the HHP technology is displayed in **(A)**. The tumor cell suspension, which has to be pressurized, has to be packed in a plastic wrapping. The pressure is applied to the treatment cavity and propagates through the pressure transmitting medium and also through the packaging (Yaldagard et al., 2008). Since the pressure is a vector product its acts on every material in the cavity with the same amplitude, independent of the shape of the probe. It was obvious in different experiments, that the pressure for inactivation should be at least equal or above 200 MPa. For the production of an effective whole cell-based vaccine, four main requirements have to be fulfilled **(B)**: the vaccine has to be safe, i.e., it has to exhibit low toxicity and, especially in the case of autologous tumor cell-based vaccines, the tumor cell material has to be effectively inactivated. The vaccine has to be further highly immunogenic, i.e., it has to trigger the immune system to elicit a strong anti-tumor response accompanied by the development of an immunological memory against the tumor. Finally, the vaccine and its processing have to be in accordance with statutory provisions.

Therefore, our group has focused on how a distinct inactivation method renders the dead tumor cells immunogenic (Weiss et al., 2010a). We examine HHP as a novel and innovative inactivation method for the generation of whole tumor cell-based vaccines. Focus is set on inactivation efficiency and the potential to deliver tumor cells with high immunogenicity. The HHP-technology is a highly reproducible technology, since pressure force vectors act orthogonal, with equal absolute value on the cell surface. Further, pressure propagation is homogenous and quasi not delayed (sound-velocitiy in media, see Figure 2A). Treatment with HHP preserves the shape of the cells and induces a gel-like consistence of cytoplasm (Frey et al., 2008). Importantly, experiments with several tumor cell lines treated with pressure equal or above 200 MPa have already proven that tumor cells are totally inactivated (Weiss et al., 2010b). So the pressurization of cells with 200 MPa leads to a mixture of apoptotic and necrotic cells (Frey et al., 2004; Korn et al., 2004; Weiss et al., 2010a). This mixture of dead cells, the cell morphology, and the observed release of danger signals after HHP treatment suggest that the tumor cells have a sufficient high enough immunogenic potential after inactivation with 200 MPa of HHP. We have demonstrated that tumor cells that were inactivated with 200 MPa HHP release DAMPs such as HMGB-1 and Hsp70 (own unpublished data). We further already revealed that the administration of HHP-treated autologous tumor cells without any adjuvant leads to a reduction of tumor outgrowth as well as to an advantage in survival in CT26 colorectal tumor bearing Balb/c mice. This was observed in a prophylactic setting (Weiss et al., 2010b). Taken together, the main prerequisites (summarized in Figure 2B) for a whole cell-based tumor vaccine are fulfilled by the application of HHP.

## Radiotherapy combined with immunotherapy for induction of anti-tumor responses

Immunotherapy provided as mono-therapy won't be successful in destroying huger tumor masses. However in combination with standard therapies that reduce the tumor burden, it can be an effective tool to obtain a prolonged anti-tumor effect. Immune surveillance diminishes the risk for the development of metastases and tumor recurrence. The combination of RT with immunotherapy therefore offers great prospects to induce a strong and prolonged anti-tumor effect.

RT is, besides CT and surgery, one of the well-established methods in clinical cancer treatment. The primary effect of RT relies on the local killing of tumor cells by damaging the DNA. However, there is growing evidence that the impact of RT is not restricted to the local destruction of tumor cells, but also on the modification of the tumor's microenvironment. It may induce abscopal effects and positively influence the systemic therapeutic outcome (Frey et al., 2012).

Solid tumors are characterized by an atypical vascular network. Additionally, tumor-associated macrophages (TAM), myeloid-derived suppressor cells (MDSC) (Marigo et al., 2008), and regulatory T-cells may foster tumor progression. They secrete chemo- and cytokines such as VEGF, IL-10, and TGF-β to suppress the maturation of DCs. They therefore hamper APCs to establish an immune response against the tumor (Melief, 2008). Since irradiation modifies the tumor cell's phenotype and the tumor microenvironment, it may contribute via bystander effects to tumor regression (Lorimore et al., 2001). It has turned out that ionizing radiation can induce pro-inflammatory conditions in the tumor by causing immunogenic cell death, associated with the release of danger signals such HMGB-1. Further, an increased macrophage activation and neutrophil infiltration has been observed after RT (Lorimore et al., 2001). Antitumor effects outside of the radiation field were already observed in numerous studies (Ehlers and Fridman, 1973; Ohba et al., 1998; Okuma et al., 2011). Originally, this phenomenon (the so called abscopal effects of X-ray) was described by Mole in 1953. Even though the molecular mechanisms are not fully known, it has become evident that the immune system plays a significant role (Mole, 1953; Demaria et al., 2004; Formenti and Demaria, 2009; Frey et al., 2012).

Due to their immunogenic features induced by e.g., RT, the dead tumor cells are rendered visible for immune surveillance. This can be regarded as an “intrinsic vaccination.” Preclinical experiments with tumor bearing mice that have been treated with RT and CTLA-4 blocking agents revealed that CTLA-4 therapy on its own is not able to elicit a systemic effect in regard to tumor outgrowth of a second, non-irradiated tumor. However, combinatory treatment with CTLA-4 blocking agents and fractionated RT resulted in a significant growth retardation of the tumor outside the radiation field (Dewan et al., 2009). Furthermore, it was observed by Schaue et al. that irradiation resulted in a dose-dependent increase of IFNγ producing T-cells that correlated with tumor control (Schaue et al., 2012).

The pivotal role of DCs for radiation-induced abscopal immune effects was demonstrated by Demaria et al. (2004). Ionizing irradiation further directly influences the ability of DCs to load antigens being associated with an improved cross-priming of T-cells (Teitz-Tennenbaum et al., 2008). Knowing that irradiation may elicit immunological responses directed against tumor cells when combined with further immune activation, the application of an additional immunotherapy to RT might be a promising approach in tumor therapy (Demaria et al., 2005). Auspicious preclinical trials examining RT in combination with cytokines (especially IL-3 and IL-12) and DC applications, have already been performed (Maraskovsky et al., 1996; Chiang et al., 1997; Lynch et al., 1997; Seetharam et al., 1999; Teitz-Tennenbaum et al., 2003; Trinchieri, 2003; Oh et al., 2004). In a rat model of glioma, the combination of a vaccine composed of irradiated glioma cells and RT was tested. Despite vaccination alone results in a reduced survival compared to the control group, the combination of RT with the vaccine was superior in regard to survival than RT alone (Graf et al., 2002). Using a mouse model of glioma, the combination of a vaccine consisting of cytokine-producing autologous cancer cell with RT was tested. This combination resulted in cure of mice; unfortunately, a group just receiving RT alone was not included in this study (Lumniczky et al., 2002).

To summarize, some combination therapies of RT and immunotherapy have already reached the level of clinical studies. As one further example, in a phase II trial for prostate cancer, the combination of standard RT and vaccination with poxvirus encoding prostate-specific antigen was examined in a two arm setting (combination versus RT only). The combination therapy provoked a cellular immune response with T-lymphocytes that were not only restricted to the TAs provided by the vaccine. This implies a radiation-induced *in vivo* immunization effect (Gulley et al., 2005).

## Outlook

The outcomes of multimodal cancer therapies including immunotherapy are complex. While treatments with antibodies are already established in the daily clinical routine, the treatment of cancer with whole cell-based vaccines is still at an experimental stage. However, recent studies revealed that such immunotherapeutic agents may broaden the anti-tumor response in cancer patients (Mellman et al., 2011). CI can help to overcome the immune suppression emanating from the tumor. Nowadays, it has become clear that the standard tumor therapies elicit local and abscopal effects (summarized in Frey et al., 2012) that could be potentiated when combined with cancer vaccines (Sistigu et al., 2011). Future research is needed focusing on which combination of standard therapies (surgery, RT, CT, RCT) with CI are most beneficial and in which tumor stage and chronology they should be applied.

### Conflict of interest statement

The authors declare that the research was conducted in the absence of any commercial or financial relationships that could be construed as a potential conflict of interest.
